# Our Correspondence

**Published:** 1869-10

**Authors:** 


### Our Correspondence.

**Letters with Answers.**

MEMPHIS, Tenn., Aug. 30, 1869.
T. S. UP DE GRAFF, M. D.,

*Dear Sir:*—I find in reading the BISTOURY that there are other folks than me who are humbugged. It is real funny to read their letters; but it doubtless opens many folks' eyes, and prevents them from being swindled in a similar manner.

That Dr. J. B. Ogden, of New York, who advertises to send a remedy *free* to all who will apply for it, is doing a "land office business" down this way. He has not only swindled me with his worthless medicine, for seminal weakness, but several of my companions also. Then there is another chap in New York who advertises in Pomeroy's *Democrat*—he calls himself J. H. REEVES, of No. 78 Nassau street. He says he has "discovered a simple means of self-cure which he will send free to his fellow sufferers.' It is the usual dodge. He sends a formula that we can't get put up anywhere, so we have to send to him for the medicine. We read your BISTOURY, and have'nt sent him any money yet, and what is more, don't mean to. I send you a club for the BISTOURY, and will increase it soon. You may publish such portions of my letter as you choose, if you think it will do anybody any good.

Yours truly, L. M.—N.

—L. M. N. tells the old story and to the point. We have no remarks to make—none being necessary.

---

BINGHAMTON, N. Y., Sept. 2, 1869.
DR. THAD. S. UP DE GRAFF,

*Dear Sir:*—I have read the letters in the BISTOURY exposing those swindlers in New York and elsewhere, and thought my experience might not be uninteresting to your many readers.

I have been a sufferer from the usual complaint, and of course have sought relief of almost every advertising "*free formula chap*" I could find. I have invariably received receipts that no intelligent druggist could put up, and have always been informed in the circular sent that the medicine could be had "already prepared of *fresh* drugs of the proprietor" for a sum varying from $3 to $15. Some I have tried, and some not, all with like effect—no good whatever.

One druggist here, though, has done a nice thing by putting up Dr. J. B. Ogden's prescription. He concluded he might as well swindle a little on his own hook as well as the doctor. So he invariably puts up some simple decoction for those who apply with the receipt, and charges from $10 to $15 for it, depending upon the price of drugs (?) and the customer's ability to pay! How's that? Is'nt that sort of swindling the swindler? Thought there was "honor among thieves!"

Yours respectfully, L——.

—Our correspondent certainly gives us a new version of the *free prescription* business. Our young men must be watchful, else they will not have the satisfaction of being swindled from headquarters. Next!

---

UPPER LISLE, N. Y., July 30, 1869.
THAD. S. UP DE GRAFF, M. D.,

*Dear Doctor:*—Inclosed I send you a club of subscribers for your delightful little Magazine. I saw one at a friend's house just in the nick of time to save me from being swindled. I sent to Mrs. M. C. Leggett, of Hoboken, N. J., for her "Indian Catarrh and Scrofula Receipt." I presented the formula to a druggist in Syracuse, who said there was no such thing as "Chee Ochee Root," and that he hadn't time to catch a "Prairie Fox" to deprive it of its "hoofs," in order to make the compound. I thought it strange that such a receipt should be sent me, but at once saw through the swindle after reading the BISTOURY.

The circular containing the "receipt" announces that "Mrs. L. will send both medicines prepared from perfectly *fresh* herbs upon receipt of $10." The other medicine is an "Indian Deafness Remedy." I have concluded not to invest, and have the BISTOURY to thank for it. I have taken real pleasure in raising this club, and mean to send you another from my own home this fall.

Very gratefully yours, MRS. H.

—We have received four letters since our last issue, complaining of the remedies (?) of this Mrs. M. C. Leggett. How persons can read her circular without seeing at once that it is intended solely to extract money from those "not posted" in business affairs, it is difficult to imagine. It bears fraud upon its very surface.

EASTON, Pa., Aug. 31, 1869.

THAD. S. UP DE GRAFF, M. D.,

EDITOR BISTOURY—*Sir* :—"I have been through the mill"—severely through—and have emerged the worst fleeced individual you ever saw. I am an "innocent" no longer, "my eye teeth are cut," and I have fully recovered from all my ills, by virtue of my madness. There is a "method in my madness," too—I'll give it you—the method consists in being fleeced, done, cheated (or something of that sort,) out of three hundred dollars, for the most abominable compounds that was ever gulped down by mortal man. Suppose I have taken enough stuff to swim a flat boat—worthless trash, too—worse than worthless—it has robbed me of what little health I once possessed.

Suffice it to say, I have patronized all those fellows who advertise to cure one *free of charge*, or at least send one receipts that will do it. Dr. John B. Ogden, Rev. Edward A. Wilson, The Howard Association, Charles J. C. Kline & Co., Prof. DaRose, Madame La Faye and the devil. I'm through—entirely through with them, and everybody like them. Why did'nt some one publish a BISTOURY and cut these fellows to pieces, long ago? I suppose I would have tried another villian—a *Reverend* one too, had I not accidentally run across the BISTOURY. Inclosed a dollar, send it to me and ———. It's the best journal I ever read. I like the way you whollop those confounded swindlers—do so some more.

Yours abstractedly,

T. W.

This letter is directly to the point. Our correspondent has written quite an essay upon quackery, without being aware of it. We will be glad to publish the experiences of others. Let us have your letters.

---

The following letter explains itself :—

PITTSBURG, Pa., July 12, 1869.

THAD. S. UP DE GRAFF, M. D.,

EDITOR BISTOURY—*Sir* :—I enclose fifty cents to pay for vol. V. of the BISTOURY. It has given me much amusement and valuable instruction—it *will* be valuable when practically required.

Please accept my thanks for those sent me heretofore.

Have you ever used bi-sulphate or sulphate of soda as an aid to quinine in fever and ague, or bilious fevers?

The surgeon at Frankford Arsenal, near Philadelphia, has used it with excellent effect for several years. He gives 10 to 15 grains before each meal.

I remark upon it as a suggestion for the BISTOURY, if it amounts to anything worthy of consideration.

Respectfully yours, J. A. K.—M. D.

